# An Automated Pipeline for Dynamic Detection of Sub-Surface Metal Loss Defects across Cold Thermography Images

**DOI:** 10.3390/s21144811

**Published:** 2021-07-14

**Authors:** Siavash Doshvarpassand, Xiangyu Wang

**Affiliations:** 1 WA School of Mines, Curtin University, Bentley, WA 6102, Australia; siavash.doshvarpassand@postgrad.curtin.edu.au; 2 School of Design and Built Environment, Curtin University, Perth, WA 6102, Australia; 3 School of Civil Engineering and Architecture, East China Jiaotong University, Nanchang 330013, China; 4 Australasian Joint Research Centre for Building Information Modelling, Curtin University, Perth, WA 6102, Australia

**Keywords:** cold-active infrared thermography, non-destructive testing, metal loss defect detection, Image processing, structural health monitoring, vision-based sensors

## Abstract

Utilising cooling stimulation as a thermal excitation means has demonstrated profound capabilities of detecting sub-surface metal loss using thermography. Previously, a prototype mechanism was introduced which accommodates a thermal camera and cooling source and operates in a reciprocating motion scanning the test piece while cold stimulation is in operation. Immediately after that, the camera registers the thermal evolution. However, thermal reflections, non-uniform stimulation and lateral heat diffusions will remain as undesirable phenomena preventing the effective observation of sub-surface defects. This becomes more challenging when there is no prior knowledge of the non-defective area in order to effectively distinguish between defective and non-defective areas. In this work, the previously automated acquisition and processing pipeline is re-designed and optimised for two purposes: 1—Through the previous work, the mentioned pipeline was used to analyse a specific area of the test piece surface in order to reconstruct the reference area and identify defects. In order to expand the application of this device over the entire test area, regardless of its extension, the pipeline is improved in which the final surface image is reconstructed by taking into account multiple segments of the test surface. The previously introduced pre-processing method of *Dynamic Reference Reconstruction* (DRR) is enhanced by using a more rigorous thresholding procedure. *Principal Component Analysis* (PCA) is then used in order for feature (DRR images) reduction. 2—The results of PCA on multiple segment images of the test surface revealed different ranges of intensities across each segment image. This potentially could cause mistaken interpretation of the defective and non-defective areas. An automated segmentation method based on *Gaussian Mixture Model* (GMM) is used to assist the expert user in more effective detection of the defective areas when the non-defective areas are uniformly characterised as background. The final results of GMM have shown not only the capability of accurately detecting subsurface metal loss as low as 37.5% but also the successful detection of defects that were either unidentifiable or invisible in either the original thermal images or their PCA transformed results.

## 1. Introduction

Corrosion is generally defined as the deterioration of the surface and internal microstructure in most metals when a reaction with a corrosive environment occurs [1,2,3,4,5,6,7]. Corrosion in its least destructive form is visible in the form of rust on the surface of metallic components. However, in its more aggressive form, it can be viewed as a loss of material along the asset’s life-cycle which can cause incremental challenges to a project’s reliability, economy and subsequent operations [1,8,9,10,11]. General corrosion detection is usually conducted in the form of determining the loss of material as the effect of corrosion activities. This yields a common yet crucial activity in corrosion mitigation, management and risk-based inspections [1,6,12,13,14].

Temperature is one of the most common identifications of the equipment and component’s health condition [11,15,16,17]. All objects at a temperature T above absolute zero (T>0 K) radiate electromagnetic radiation. Mentioned radiation is classified as infrared bandwidth along the electromagnetic spectrum (wavelength in the range of 0.75–1000 μm). Infrared thermography, thermal imaging or, in general, thermography is considered an NDE practice that allows us to perceive heat waves on an object’s surface [18].

Active thermography is a technique benefited by an external excitation source for the purpose of stimulating the thermal evolution inside the object. This energy source can be either physical (stimulating internal energy by causing internal vibration e.g., microwave or electromagnetic Eddy current) or optical (e.g., heating or cooling methods). Infrared thermography devices register both the temporal and spatial evolution of surface temperature from the stimulation moment till stabilising to the ambient state.

**Figure 1:**
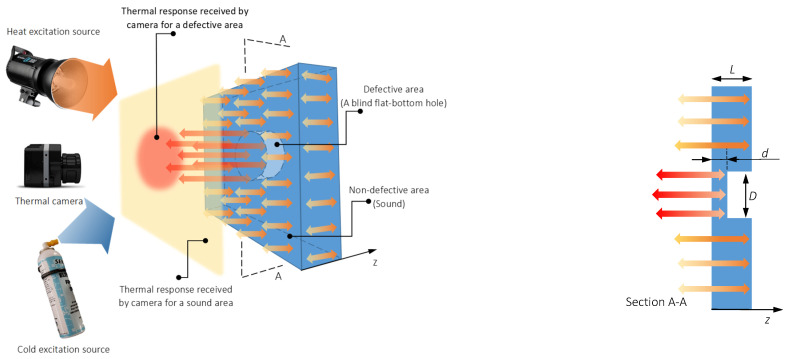
Schematic of thermal diffusion and response through a defective solid [1] Reproduced with permission from Infrared Physics & Technology, Elsevier, 2019. Licence number 5106340697590.

The physical foundation of defect detection using active thermography is based on the thermal conduction (diffusion) phenomena in solids. Solving the thermal wave equation for certain boundary conditions results in estimating important information e.g., temporal temperature evolution. The classical model of heat conduction, known as the Fourier equation, is as follows [19]:

(1) $$\frac{\partial T}{\partial t}=\delta {▽}^{2}T$$

where δ=k/ρC is thermal diffusivity $\left(m^{2}/s\right)$. k is thermal conductivity (W/mK); ρ is density $\left(\mathrm{kg}/m^{3}\right)$ and C is specific heat (J/kg K). Sub-surface defects will prevent the diffusion of stimulation energy propagated inside objects. As a result, the defects will appear in the image in the form of anomalies with different contrasts compared to the surrounding sound (non-defective) area, see Figure 1.

Thermal diffusion mechanisms for heating and cooling are similar and both follow the Fourier equation, see Equation (1). As a result, there has been some recent interest in investigating the use of cooling mediums instead of heating processes as an excitation mechanism [2].

Defect detection via active *pulse thermography* (PT) experiments consists of a specimen subjected to a relatively short energy pulse and then recording the temperature evolution curves in transient mode (temporal). The thermal (hot or cold) front is distributed under the surface based on the diffusion phenomena, see Equation (1).

As defects located at different depths diverge the diffusion rate of the energy pulse differently, each defect will appear at a different time to the other defects [20,21]. In practice, producing the ideal Dirac pulse (unit-area pulse) is not possible. As a result, the square pulse of width t and amplitude A accounts for the most practised waveform in PT applications [22,23]. However, the pulse width can be varied from an approximate ~2 ms (quasi-Dirac pulse) to a couple of seconds (Long pulse) [1].

Consideration of a non-defective reference area and the calculation of the temperature difference of a spatial domain over a temporal domain to enhance subsurface defect visibility has been a hugely practised procedure in the literature [24,25,26]. However, in a real-life case of corrosion (metal loss), identifying a non-defective or reference may not be always possible. In order to obviate this need, a method known as *Dynamic Reference Reconstruction* (DRR) was introduced [2]. The motivation behind this method was the relative coexistence of reference (non-defective) and defective areas in each other’s proximity; meaning a non-defective area can be considered as defective based on its contrast intensity level or, in contrast, a defective area can be hidden in a non-defective surrounding due to the high similarity of their intensities. In previous work [2], *Adaptive Histogram Equalisation* (AHE) method for contrast enhancement was dynamically applied to a block of pixels moving across a selected segment of time–transient thermography images of an artificially defective test piece.

## 2. Materials and Methods

**Figure 2:**
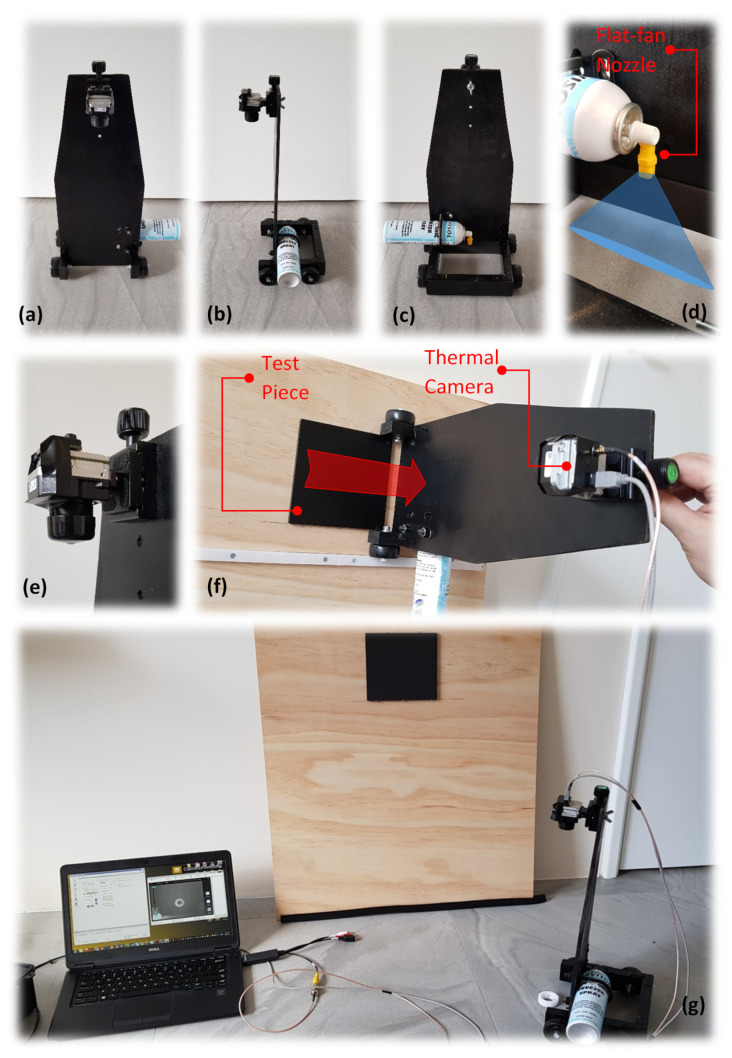
(**a**) carrier front view; (**b**) carrier side view; (**c**) carrier back view; (**d**) cooling medium spray with customised flat-fan nozzle; (**e**) thermal camera; (**f**) carrier linear movement over the test piece; (**g**) experimental setup [2], reproduced with permission from authors own work, *Sub-surface metal loss defect detection using cold thermography and dynamic reference reconstruction (DRR).* Structural Health Monitoring, 2021: p. 1475921721999599.

In conjunction with the previous work [2], experimenting the potential usage of cold long pulse (1 or 2 s cold burst) as the stimulation source is of interest. A commercial cooling medium known as freezing spray is found to be sufficiently accessible and practical. A prototype mechanism consisting of a carrier accommodating both the thermal camera and the cooling spray reservoir (can) is manufactured, see Figure 2. The component’s arrangement has been configured in a way that the spraying action can be manually operated in full blast above the test surface while the carrier passes over the test piece surface immediately exposing the stimulated surface to the camera, see Figure 2f. A barrier which holds the camera prevents escaping cold burst (noise) to disrupt the camera view. The camera is connected to a computer via an analogue cable transferring the video signals to recording software, see Figure 2g. A test frame is used in order to accommodate the test piece in a fixed position while incorporating a guide rail into the test frame, ensuring the carrier’s linear motion across the test specimen, see Figure 2f. In order to prevent non-uniform cooling of the surface, a modified flat-fan jet spray nozzle is used. The flat-fan jet nozzle sprays the cold burst from a very narrow slot creating a quasi-linear cross-section at the specimen’s surface contact, see Figure 2d.

The thermal device used for the purpose of this work is an uncooled LWIR (Long Wave Infra-Red) FLIR TAU2 640 thermal camera characterised with 640 × 512 pixels output resolution, 17 μm pixel size and the 7.5–13.5 μm spectral operating band. This device is equipped with a 19 mm WFOV (Wide Field of View) lens with 32°×28° (h×v). A 19 mm lens is found to be optimum in terms of providing relatively wide coverage of the surface with the minimum image distortion. TAU 2 640 is capable of recording video signals at 30 Hz nominal rate (29.97 Hz NTSC and 25 Hz PAL) in both 8 bit (analogue) an 14 bit (digital) colour levels. Experiments are performed under ambient temperature and pressure.

**Figure 3:**
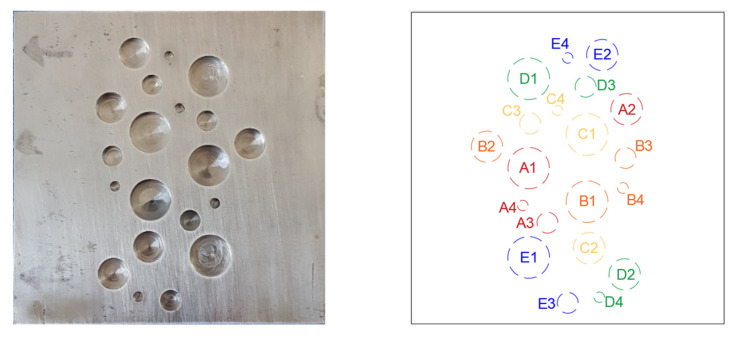
The test piece (hidden and exposed surfaces) and the distribution of artificial defects across its hidden surface.

The test piece is manufactured from AISI 1018 mild carbon steel (See material properties in Table 1) with the dimensions of 150 × 150 × 8 mm. Various sizes and depths of flat bottom circular blind holes are randomly distributed across one side (hidden side) in order to replicate the random metal loss defects, see Figure 3 and Table 2. As shown in Figure 2, the test piece surface is covered by low sheen black paint. This will reduce the possible external reflection from the surroundings. However, we found that cold stimulation using a cooling medium is less prone to create reflections on a bare metal surface in comparison with heat pulse sources e.g., flash lamps.

In this work, the previously automated acquisition and processing pipeline is re-designed in order to: (1) Expand the application of the device over the entire test area. This can ideally create a continuous scan of the test surface for potential in-situ applications. In order to cover an extensive surface of the test piece, it is necessary to take into account multiple segments of the test surface scanned by the device. The previously introduced pre-processing method of *Dynamic Reference Reconstruction* (DRR) is enhanced by using a more rigorous thresholding procedure combined with *Principal Component Analysis* (PCA) in order for feature (DRR images) reduction; (2) Introduce an automated segmentation method based on *Gaussian Mixture Model* (GMM) in order to effective detection of defective areas when the non-defective areas are uniformly characterised as background.

## 3. Results and Discussions

**Figure 4:**
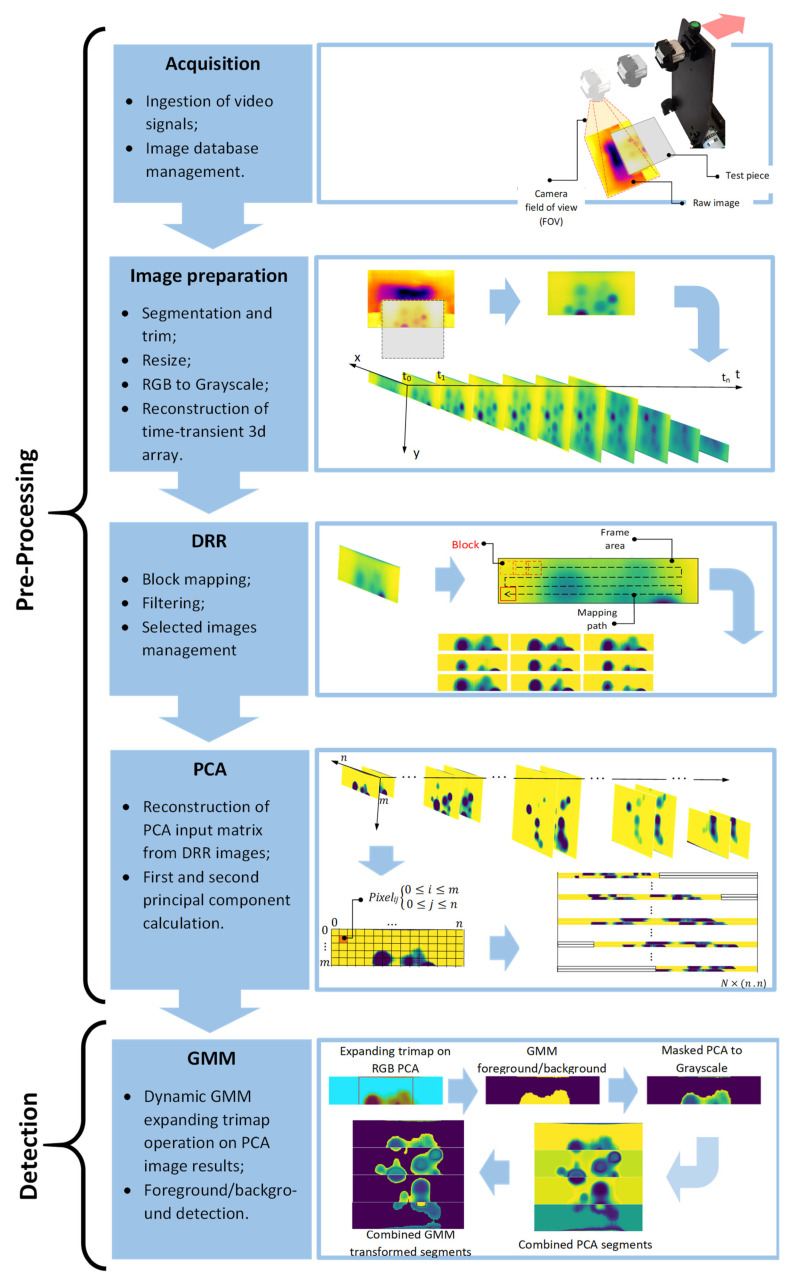
The automated data acquisition and analytics pipeline steps.

A comprehensive automated image data acquisition, pre-processing and analytics pipeline was designed. Python programming language was used for scripting and prototyping. Various steps of the mentioned pipeline are shown in Figure 4. Those include: (1), the acquisition of video signals captured by a thermal camera; (2) decomposing the video to frames, trimming (crop) each frame to only reveal the test piece exposed surface to the cold burst and stacking all frames in order to produce a 3D array of frames over the exposure time; (3) applying DRR by mapping a block of pixels, generating the contrast-enhanced images by applying *Adaptive Histogram Equalisation* (AHE) and filtering the enhanced image based on a multi-boundary condition on block location; (4) applying PCA on equal size segments of DRR images by reconstructing the PCA input matrix; and finally (5) applying an iterative “GrabCut” algorithm on first and second principal components of each segment.

### 3.1. Image Data Pre-Processing

The experimental configuration consists of both the manual operation of the cooling spray and the manual travelling of the carrier while a cooling burst is in action. As result, the assurance of constant linear travelling speed in order to guaranty the maximum uniform cooling as well as equal exposure of the test piece surface to the thermal camera is paramount.

**Figure 5:**
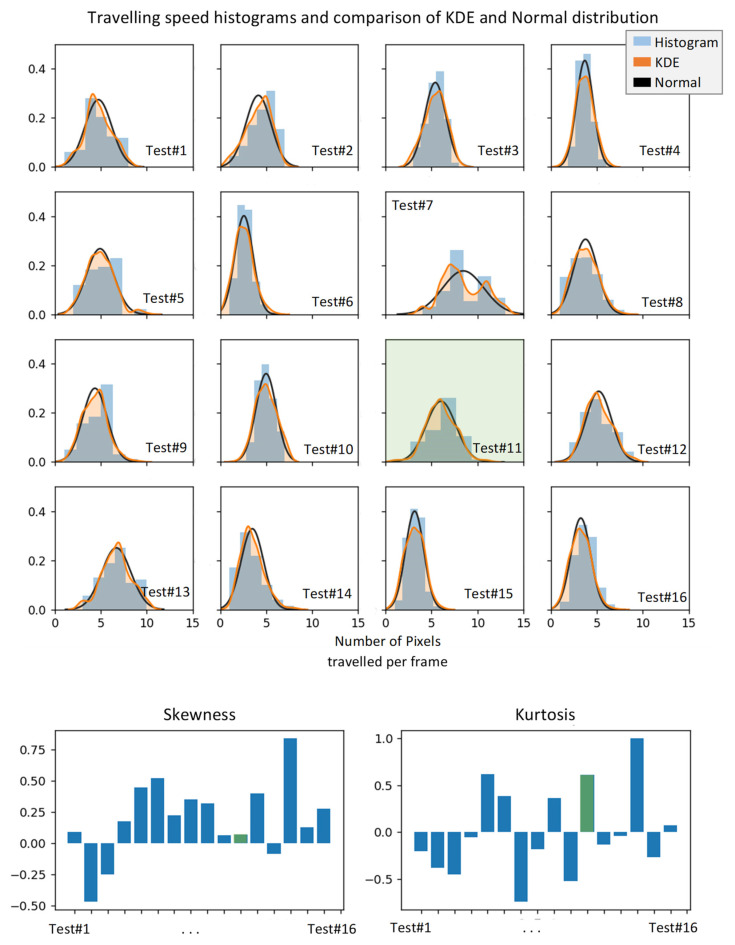
Statistical parameters representing the uniformity and consistency of the carrier speed.

Sixteen experiments were conducted. Histogram and *Kernel Distribution Estimation* function (KDE) of carrier speed in pixel per frame unit for each test was used to select the most acceptable test conditions and results, see Figure 5. The measure of the most acceptable test condition under constant carrier speed was considered as the least skewed (skewness = 0) and the highest positive kurtosis (kurtosis >> 0) between tests histograms. Skewness is the measure of dataset symmetry or lack of symmetry with skewness = 0 defines the normal distribution. Kurtosis is the measure of dataset sharpness with positive kurtosis and represents the sharp (unique) distribution of the dataset, negative kurtosis represent the flat (uniform) distribution of the dataset and zero kurtosis corresponds to the normal distribution (based on Fisher definition). From a collection of 16 tests, the results of test number 11 are selected to be considered for defect detection processing. Test number 11 demonstrated that its KDE is very similar to its ideal normal distribution. From this statistical analysis, the carrier travelling speed was estimated at about 6 pixel/frame. For a raw image of 450×450 pixel resolution, this translates to 180 pixel/second which is the equivalent to 60 mm/sec for the test piece. As a result, a complete scan of the 150 × 150 mm test piece was performed in approximately 2.5 s.

Image contrast enhancement methods use various transformations (mapping functions) to map the existing pixel intensity values to a new set of intensity values [27,28]. Depending on the transformation function deployed, contrast enhancement procedures can be divided into linear and non-linear transformations. *Global Histogram Equalization* (GHE) benefits from non-linear transformation.

**Figure 6:**
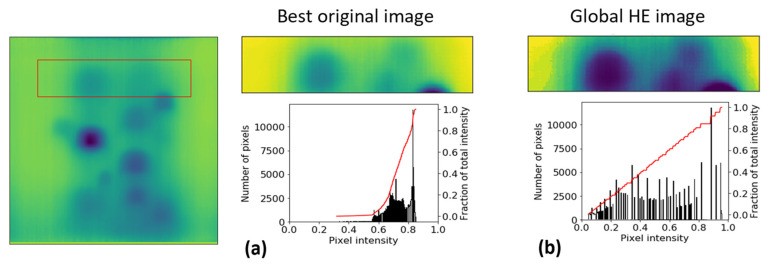
Comparison of the original image and globally equalised histogram image and their histogram (black) and cumulative distribution function (red) for a selected area of the test surface (**a**,**b**) [2], reproduced with permission from authors own work, *Sub-surface metal loss defect detection using cold thermography and dynamic reference reconstruction (DRR).* Structural Health Monitoring, 2021: p. 1475921721999599.

For a discrete condition, the probability of a pixel intensity occurrence of level r in an original image of size M×N is:

(2) $$P\left(r\right)=\frac{number\;of\;pixels\;with\;intensity\;level\;“r”}{M.N}\;r=0,\;1,\;\ldots ,\;\left(L-1\right)$$

where, L corresponds to the number of grey levels presented in the original image (for an 8-bit grayscale image = 256 which can be normalised to intensity values between 0 and 1). Here, creating a transformation function of the form s=T(r) which generates a new image with a flat (equalised or uniform) intensity histogram is of interest. The resulting image would have a linearised *Cumulative Distribution Function* (CDF) across the intensity value range meaning the fraction of each intensity level is almost equal, see Figure 6. In fact, for discrete conditions, the CDF represents the cumulative values of the PDF or:

(3) $$C\left(r\right)=\overset{r}{\underset{i=0}{\sum }}P\left(i\right)$$

Eventually, the relationship between the intensity value of a pixel in the original image, r, and its new intensity value, s, in the enhanced image is [29,30]:

(4) $$s=\frac{\left(L-1\right)}{M.N}T\left(r\right)=\frac{\left(L-1\right)}{M.N}\overset{r}{\underset{i=0}{\sum }}P\left(i\right)=\frac{\left(L-1\right)}{M.N}C\left(r\right)$$

**Figure 7:**
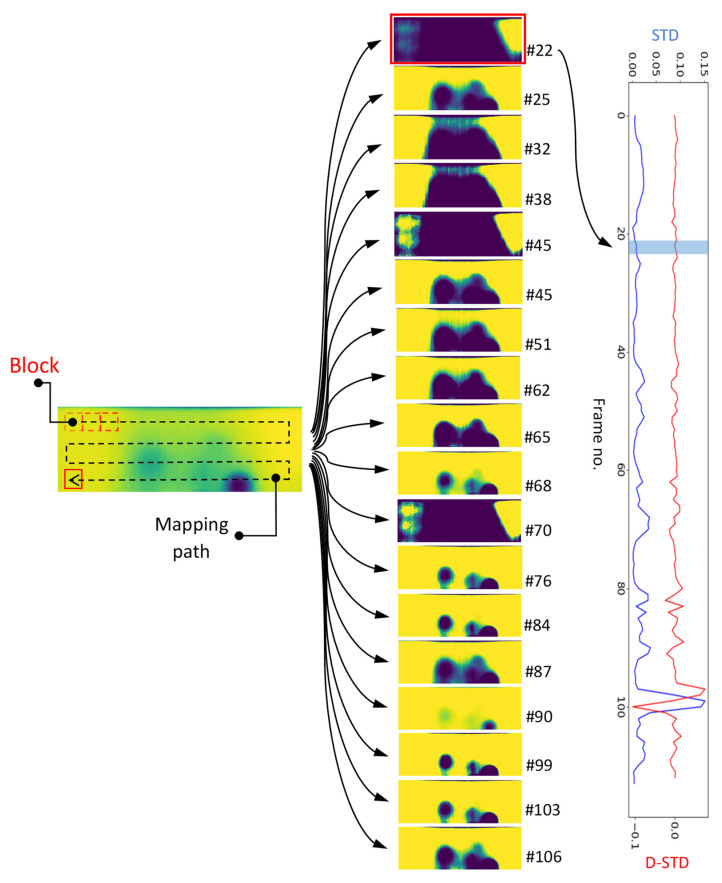
An example of a pre-processed original thermal image subject to a 40×40 mapping block and its DRR selected images based on block standard deviation threshold.

Some of global histogram equalisation issues e.g., over/under-enhancement of defects versus surroundings causing blocking effects, see Figure 6, led to more attention on deploying the AHE method on thermal images [2]. In AHE, each pixel intensity level is transformed exactly similar to the global histogram equalization, see Equation (4). However, the derivation of the transformation function for each pixel is proportional to the CDF of pixel values in the block (kernel) of surrounding pixels of size $M_{b}\times N_{b}$ and not the entire image [31]. Nevertheless, both GHE and AHE enhanced images have shown some undesirable over/under enhancing effects [2]. In fact, the lack of prior knowledge of non-defective areas prevents the traditional image enhancement methods e.g., GHE and AHE to distinguish the defective area from non-defective areas due to the effects of non-uniform stimulation or energy lateral diffusion. Also, the performance of other traditional thermal time-transient image processing methods e.g., thermal contrasting and Fourier transformation, still require identification of reference areas even if they are applicable (here the stimulation waveform is considered a long pulse). DRR was introduced in order to dynamically reconstruct AHE transformed images in which the presence of reference and defects in the image is less affected by over/under enhanced artifacts caused by non-uniform cooling, lateral diffusion, etc. [2]. This was done by first, deploying AHE transformation of the original image based on the intensity of mapped blocks of pixels moving across each image frame. Then the redundant (over/under enhanced) images are filtered by selecting only the enhanced images when the mapping block was passing over a certain location across the original image. This certain location is found to be very effective in reconstructing the reference against defect areas when the block is at the transition boundary between very low (dark) intensity pixels and very high (light) intensity pixels [2]. In previous work, standard deviation of the blocks pixel intensities distribution was chosen as the indicator of block location with local maximum standard deviations showing a transition between low and high contrast areas [2]. Here, we improved the filtering process by considering a more rigorous boundary condition associated with the reference/defect transition boundary. Figure 7 represents one of the pre-processed original frames and the selected images from deploying DRR with a standard deviation of the block as the sole measure of transition location. The size of the block is 40×40 pixels. As shown in Figure 7, from 114 block locations along the mapping path, 18 DRR enhanced images are selected. However, not all these images are optimally selected based on a local maximum standard deviation thresholding scheme. For instance, looking at image frame number 22, one can observe that this image is highly over-enhanced with a major portion of low (dark) intensities covering the defects. However, this image is selected as the mapping block location as this frame has satisfied the filtering threshold.

**Figure 8:**
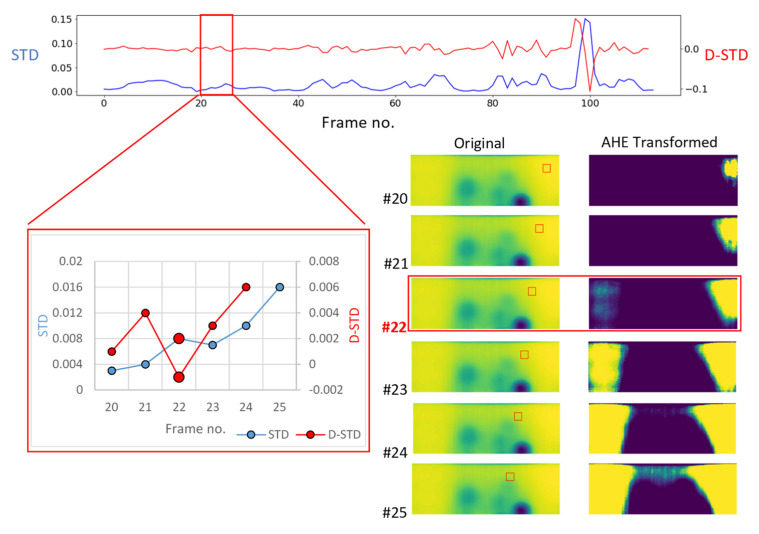
Trend of STD and D-STD mapping block transitioning at frame #22.

Figure 8 represents the mapping path and block movement sequences resulting in the frame 22 as well as standard deviation (STD) and derivative of standard deviation (D-STD) trend for before and after the mentioned frame. In frame 22, the block is transitioning from a reference area to another reference area with different intensities caused by a non-uniform cooling burst or possible thermal lateral diffusion. This is sufficient to mislead the DRR filtering process by selecting frame 22 as it perfectly satisfies the filtering condition.

In order to improve the filtering process and prevent redundant AHE transformed frames such as frame 22 to be selected, three additional sets of filtering thresholds were added:

(5) $$\wedge \{\begin{array}{c} \overset{´}{\sigma_{b}}\left[i\right]<0\;\;\left(previously\;added\;threshold\right)\\ \;\overset{´}{\sigma_{b}}\left[i-1\right]>0\;\left(previously\;added\;threshold\right)\;\\ \mu_{b}\left[i+1\right]\leq \;\mu_{b}\left[i\right]\;\leq \mu_{b}\left[i-1\right]\;\left(newly\;added\;threshold\right)\\ \sigma_{b}\left[i\right]>Q_{2}\left(\sigma_{b}|\begin{array}{c} k\\ 0 \end{array}\right)\;\left(newly\;added\;threshold\right)\\ \mu_{b}\left[i\right]<Q_{2}\left(\mu_{b}|\begin{array}{c} k\\ 0 \end{array}\right)\;\left(newly\;added\;threshold\right) \end{array}$$

where, index b corresponds to a block value; σ represents standard deviation of the block intensities; $\sigma^{\prime }$ is the derivative of standard deviation of blocks intensities; μ corresponds to block mean intensity value; $Q_{2}$ represents the second quartile (median) of a parameter across all blocks; i is the location of the block along the mapping path and k is the number of possible blocks being mapped on each original thermal image. It is important to stress, ∧ (and) logical operand connects all five above thresholds meaning they all need to be satisfied in order to select an image as DRR enhanced image. The above threshold combination was used for a block transitioning from a high intensity (light colour—potentially a reference area) to a lower intensity (dark colour—potentially a defective area). With a minor change to Equation (5), the thresholding combination can be modified for a block when transitioning from low intensities (dark) areas to high intensities (light) areas.

(6) $$\wedge \{\begin{array}{c} \overset{´}{\sigma_{b}}\left[i\right]<0\;\;\left(previously\;added\;threshold\right)\\ \;\overset{´}{\sigma_{b}}\left[i-1\right]>0\;\left(previously\;added\;threshold\right)\;\\ \mu_{b}\left[i+1\right]\geq \;\mu_{b}\left[i\right]\;\geq \mu_{b}\left[i-1\right]\;\left(newly\;added\;threshold\right)\\ \sigma_{b}\left[i\right]>Q_{2}\left(\sigma_{b}|\begin{array}{c} k\\ 0 \end{array}\right)\;\left(newly\;added\;threshold\right)\\ \mu_{b}\left[i\right]<Q_{2}\left(\mu_{b}|\begin{array}{c} k\\ 0 \end{array}\right)\;\left(newly\;added\;threshold\right) \end{array}$$

**Figure 9:**
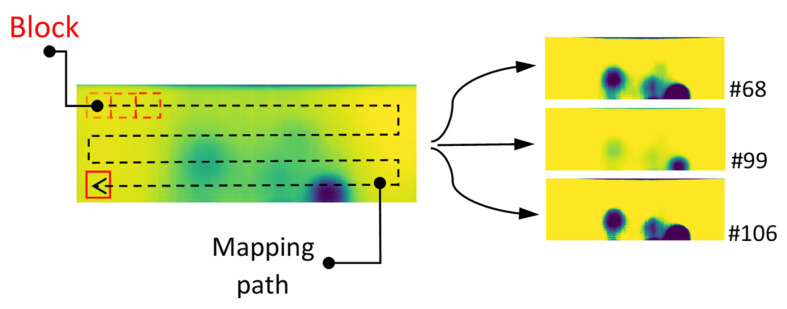
Improved DRR enhanced image selection across the Figure 8 original image.

Figure 9 represents the DRR selected frames of the same original image shown in Figure 7. By deploying the new sets of thresholds in order to filter the redundant frames, a significant improvement was achieved in order to prevent more redundant transformed frames to be selected. As part of the image data complexity, dealing with a high volume of images generated through a DRR process can be challenging. In fact, the mentioned improved filtering (thresholding) scheme significantly improved the outcome of the next step in the pipeline, which is PCA, by not only reducing the number of redundant images but also by significantly reducing the computational time of performing PCA on DRR selected images. From 4483 AHE transformed images of entire time-transient frames of the test surface, 810 images were selected by DRR.

Selected DRR images need to be reconstructed to one single image representing all enhanced defects and references. DRR images carry both spatial and temporal information pixels. There are a variety of feature reduction (dimensionality reduction) methods used in the literature, however, PCA is one of the most practised methods in the thermography community [2,32,33]. One reason to prefer PCA over other statistical methods of feature reduction e.g., LDA, Random Forest, etc. is the unsupervised nature of PCA, meaning there is no need to define classes of features (here different intensities of pixels). PCA is a method based on the singular value decomposition (SVD). For the purpose of thermal images processing, this can be applied to the measured temperature (intensity) signature of each pixel in the image frame sequences [2,34]. This technique reduces the dimensionality of thermal data from 3 dimensions (spatial and temporal) to 2 dimensions. This is done by reconstructing the input data (time-transient images) in a matrix in which each row consists of a raster-like (flattened) arrangement of all pixels in every single image. As a result, each column in the input matrix represents the temporal evolution of each individual pixel. Then, using eigenvalues and eigenvectors as the key by-products of SVD, the input data (reconstructed matrix) will be decomposed to its empirical orthogonal functions (EOF).

**Figure 10:**
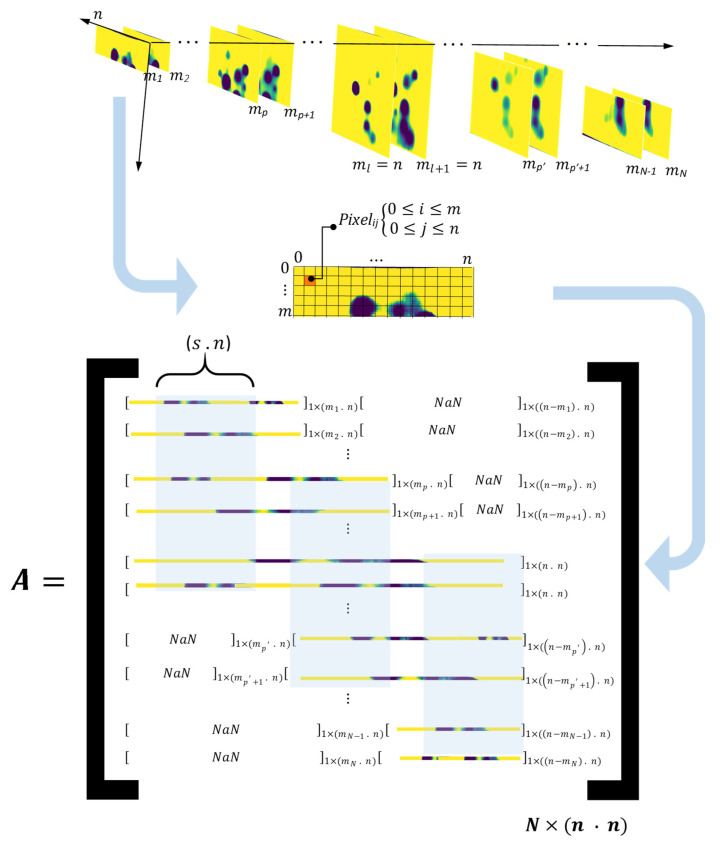
Generating the PCA input matrix from time-transient 3D array of DRR selected images.

If we assume matrix A as the rearranged input data then:

(7) $$S=\left(A-A_{mean}\right){\left(A-A_{mean}\right)}^{T}=U\Gamma U^{T}$$

where S is the covariance matrix; U corresponds to the matrix containing the eigenvectors of $S=\left(A-A_{mean}\right){\left(A-A_{mean}\right)}^{T}$. Also, Γ is a diagonal matrix containing the singular values (non-negative square root) of $S^{T}={\left(A-A_{mean}\right)}^{T}\left(A-A_{mean}\right)$ eigenvalues. Figure 10 represents the procedure of reconstructing the PCA input matrix from DRR selected images. Each DRR image of size $\left(m_{l}\times n\right)$ with l=(1, 2, …, N) where N is equivalent to the number of DRR images, is restructured (flattened) to a $1\times \left(m_{l}\;.n\right)$ one dimensional array. This array then is assigned respectively to a row in input matrix A. Considering the different sizes of DRR images generated, the size of the final input matrix will be N×(n . n) where n is the maximum size of a square DRR image which is the equivalent to the maximum field of view that the thermal camera can receive from a test piece surface. As incorporating the thermal evolution of each pixel in order to conduct PCA is paramount, a test piece surface can be divided into multiple equal segments of size s×n. This will allow the participation of all DRR images from the moment that segment s enters the camera’s field of view until it leaves the field of view. In other words, the input matrix A is equally sliced by seg.

**Figure 11:**
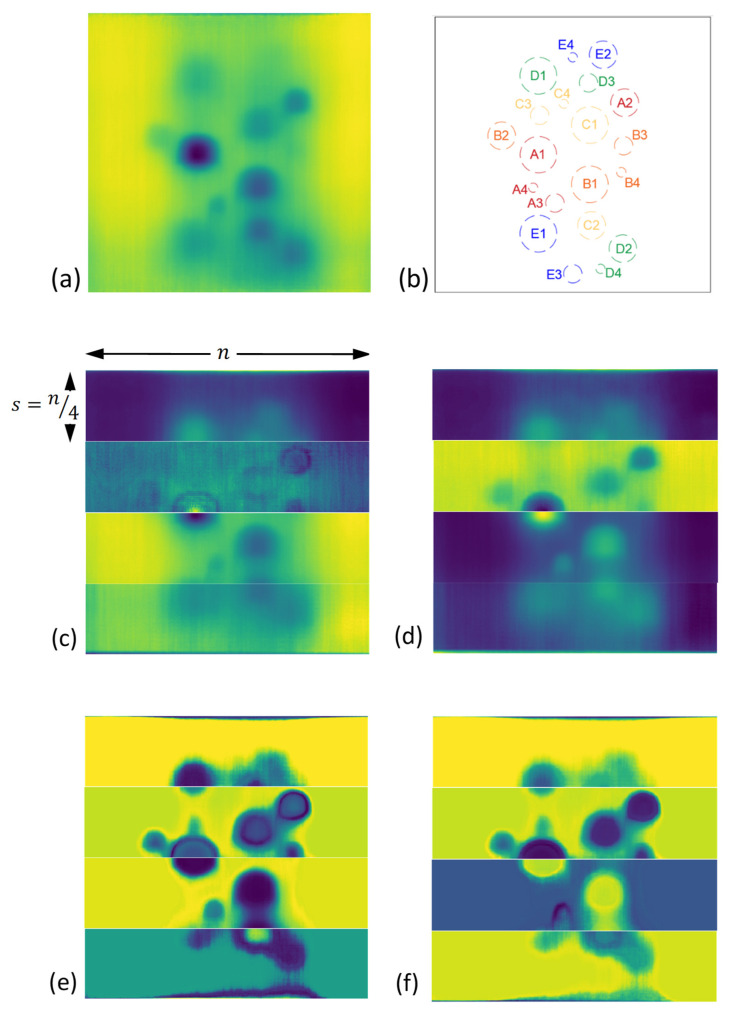
(**a**) the test piece original full-frame thermal image; (**b**) defects schematic; (**c**,**d**) the first and second PCA of original time-transient thermal images; (**e**,**f**) the first and second PCA of time-transient DRR selected images.

After performing PCA on equal segments, each PCA by-product vector is reshaped into its original 2D array (image) of size (s×n). Figure 11 demonstrates the PCA result for four segments of the test piece surface compared to the result of PCA on originally captured thermal images.

### 3.2. Detection

Despite the highly improved results of PCA on the DRR selected images, each generated PCA image segment can obtain a different intensity distribution, see Figure 11. This results in diversity in the intensity schemes, especially in the reference (non-defective) areas where they can appear with different colour intensities in each of the segments. This requires human expert judgment in order to interpret images and identify the defects. In order to automate the process to identify the defects as autonomously as possible, a foreground/background detection (segmentation) algorithm known as GrabCut [35,36] with a modification is used. The traditional GrabCut segmentation steps are as follow:

- User initialises a labelled sample of pixels known as trimap T in an RGB image with color intensities of $z=\left(z_{1},\;\cdots ,\;z_{n},\;\cdots ,\;z_{N}\right)$ and transparency of $\alpha =\left(\alpha_{1},\;\cdots ,\;\alpha_{n},\;\cdots ,\;\alpha_{N}\right)$. This trimap can be either in the form of a manual identification (highlighting) the background/foreground in the original image or selecting an area fully surrounding the foreground, meaning all the pixels out of this area (generally a rectangle) are labelled as definite background. Here, the labelling follows the context of “hard” labelling meaning $\alpha_{n}\in \left\{0,1\right\}$ where any pixel in the background will be labeled as 0 and any pixel in the foreground will be labeled as 1. In this work, the concept of rectangle trimap is used where only the labelled background pixels are supplied as $\alpha_{n}=0$ for $n\in T_{B}$ (background) and any pixel inside the trimap is assigned to $T_{U}$ (undefined).
- For each background (definite background) and undefined (probable background or foreground) pixel set a Gaussian distribution function θ is initialised. *Gaussian Mixture* is used to model each colour channels distribution, each identified by k∈{1, …, K}, where K is the number of classes in the data which here is equivalent to 2 (background or foreground) [35,36,37]. In order to find the optimum parameters of each distinct distribution function along the D-dimensional data (D=3, for a RGB image), an *expectation-maximization* algorithm is used. EM is an iterative method to find maximum likelihood or maximum *a posteriori* estimates of distribution parameters, where the model depends on unobserved latent parameters. These parameters are θ={π(α, k), μ(α, k), Σ(α, k), α=0,1, k=1, …, K} and the Gaussian distribution can be written as:

(8) $$\theta \left(z|\mu ,\Sigma \right)=\frac{1}{{\left(2\pi \right)}^{D/2}{\left|\Sigma \right|}^{1/2}}exp\left(-\frac{1}{2}{\left(z-\mu \right)}^{T}\Sigma^{-1}\left(z-\mu \right)\right)$$

where π corresponds to mixing probability or mixture weighting coefficient, meaning how probable it is for a data point $z_{n}$ to be a member of Gaussian distribution $\theta_{k}$, and $\overset{K}{\underset{k=1}{\sum }}\pi_{k}=1$. μ corresponds to the distribution mean which defines the centre of cluster data and Σ is equivalent to the covariance of the distribution which represents the width of the distribution for the 2D Gaussian components for the background and foreground distributions.

After assigning each pixel of $n\in T_{U}$ to either a background or foreground distribution, a segmentation operation is done by defining an energy function E so that its minimum should correspond to a successful segmentation [35,38]. This energy function is known as *Gibbs* energy and comprises two terms:

(9) E(α, k, θ, z)=U(α, k, θ, z)+V(α, z)

where U, the data term, evaluates the fit of the transparency distribution α to the data z, given the distribution model, θ. V, the smoothness term, measures how smooth the labelling is over neighbouring pixels [38].

**Figure 12:**
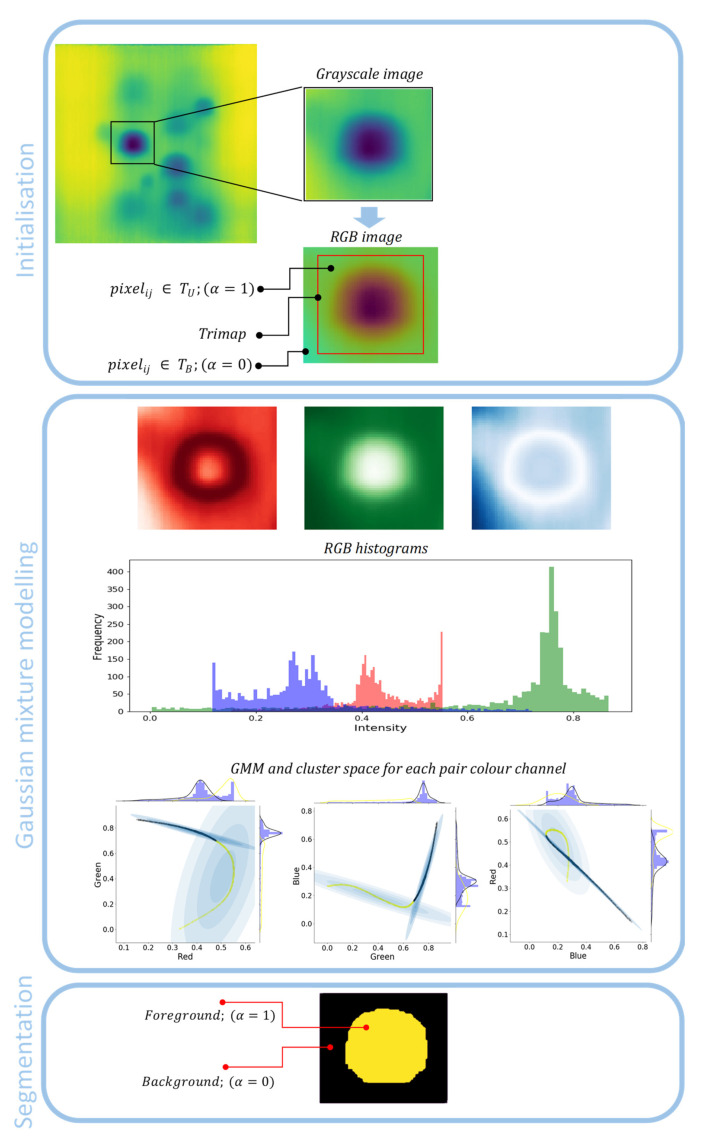
Three steps of defect segmentation using GrabCut algorithm.

Figure 12 represents an example of the segmentation process for a selected area in one of the original thermal images. This area includes a defect and its surrounding pixels. Here, successfully segmenting the defect from its background is paramount. As shown in the Gaussian mixture modelling section, the Gaussian mixture model clusters the pixel data for each pair of colour channels based on two initially defined classes of background (blacks) and foregrounds (yellows). In fact, each colour channel intensity histogram is modelled by two Gaussian distributions for background and foreground and the ellipses demonstrate each cluster space.

**Figure 13:**
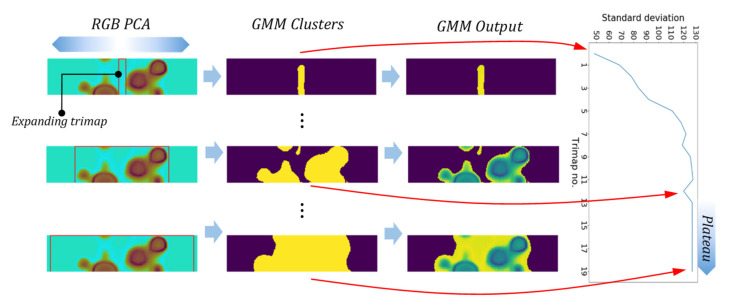
Example of expanding trimap operation and the segmentation convergence criteria.

**Figure 14:**
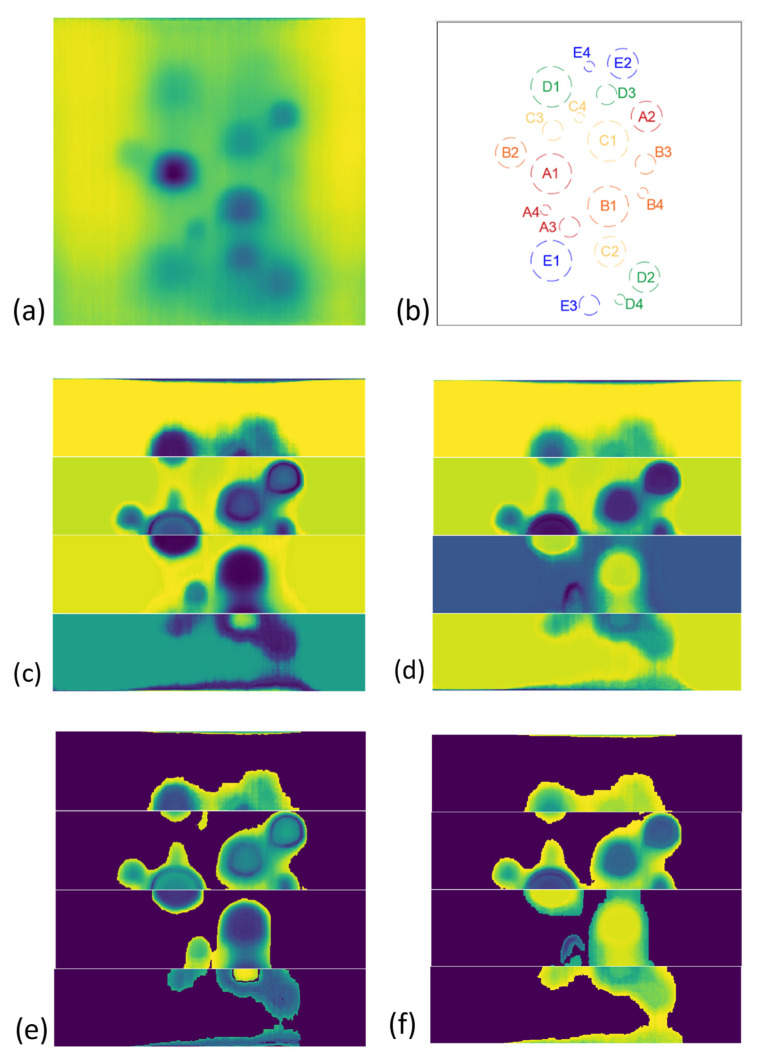
(**a**) the test piece original full-frame thermal image; (**b**) defects schematic; (**c**,**d**) the first and second PCA of time-transient DRR selected images; (**e**,**f**) respective GMM segmented PCA images.

Selecting a right trimap location covering the entire probable foreground, however, remains a challenge that requires user supervision and prior knowledge of existing foregrounds and backgrounds in the image. In order to resolve this issue, we proposed an automated unsupervised trimap assignment to the PCA images. As shown in Figure 12, the correct selection of trimap (rectangle) is quite crucial in order for the GMM’s successful training. Here an expanding rectangular trimap was designed to iteratively expand over each PCA image segment. A convergence criterion was defined based on the total standard deviation of each GMM cluster of images. When the standard deviation plateaus the trimap expansion does not generate a different segmentation than the previous trimap, see Figure 13. This will trigger the selection of the correct trimap. Figure 14 represents the final results of GMM segmentation on PCA images. A significant part of the test piece is correctly identified as a reference area with enhanced visibility of defects which required minimum human interaction along the process.

Table 3 represents a comparison between the defect visibility statuses across original thermal images, PCA of original thermal images, PCA of DRR images and the detection status of defects in the GMM images. In fact, not only the visibility of some defects i.e., D1, D2, E1 and E2 has been significantly improved compared to the original PCA results but also some invisible defects in both the original image as well as PCA of the original image i.e., B3, C3 and D3, can be identified in the DRR PCA images. In general, this pipeline has shown very profound success in removing the blocking effects of some unavoidable non-uniform cooling (when comparing the non-defective area at the centreline of the defect compared to the non-defective area located at the sidelines. The defects as low as 5 mm diameter, were not able to be detected regardless of their depth. Despite highly improved results, there is still a minor portion of the actual reference areas which is identified as defective. Especially the areas between two or more adjacent defects in which there is high susceptibility to lateral energy diffusion.

It is worthwhile comparing the performance of defect detection using cooling stimulation and heating stimulation. However, the nature of the current experimental setup, including the use of long-pulse excitation in a dynamic setup, has made such comparisons challenging. The majority of research involved with defect detection in highly conductive materials has benefited from using pulsed thermography. For this comparison, the unprocessed (raw) and initial results of this work were compared with the results of a work performed by Almond et al. [39]. Mentioned work results are selected for this comparison based on the following considerations:

First, the Almond et al. experiment is one of the few works in the body of literature which has been performed based on the long pulse heating excitation waveform (~5 s). In the current work, the cooling stimulation operates in long pulse mode scanning the entire surface in ~2.5 s.

Second, in the test piece in the Almond et al. experiment it includes a 6 mm thickness mild steel plate with 10, 15 and 20 mm diameter defects of 4 to 0.5 mm depth from the surface. This translates to as low as 33.3% metal loss (for 4 mm depth defect). In our work, the test piece consists of an 8 mm thickness mild steel plate 5, 10, 15 and 20 mm diameter defects from 5 to 1 mm depth from the surface. This translates to as low as 37.5% metal loss (for 5 mm depth defects).

Lastly, the Almond et al. test was performed using a 60 Hz sampling rate thermal camera which is not significantly higher than 30 Hz sampling rate in the current work.

**Figure 15:**
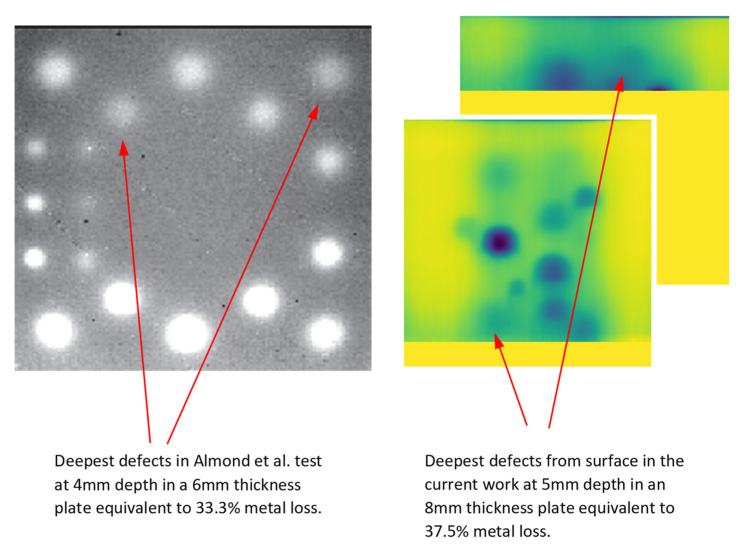
A comparison of unprocessed (raw) images of defects in mild steel using long heating pulse versus cooling excitation. Left represents a 6 mm thickness mild-steel plate subjected to 5 s heat pulse [37]. Right represents the results of current work comprising an 8 mm thickness mild-steel subjected to cold burst and registered immediately after being revealed to the camera. Reproduced with permission from Almond et al., Long pulse excitation thermographic non-destructive evaluation; published by NDT&E International, Elsevier, 2017. Licence number 5095160469715.

Figure 15 shows that using cooling stimulation (long pulse) as the excitation source is characterised with an almost similar capability in defect detection in comparison with heating long pulse. It is, however, important to stress that the defect distribution across the test specimen in the current work has been intentionally designed towards keeping the defects in close proximity to each other in order to not diminish the effect of lateral diffusions.

## 4. Conclusions

In this work, the previously introduced pre-processing method known as DRR was improved by using a more rigorous thresholding procedure. This resulted in improving both image data quantity (reduced number of images) and quality (reduced redundant images). Further, an automated segmentation method based on GMM assisted the expert user with more effective and autonomous detection of the defective areas across reconstructed reference areas. The visibility and detection status of defects were compared using both PCA of original thermal images against PCA of DRR images. A significant improvement in visibility and detection of some unidentifiable (difficult to separate the defect from the surroundings) and invisible defects was achieved.

Active IRT using cooling medium demonstrated a sound capability to be implemented as an alternative to a condition monitoring technique using heating devices. The final outcome of the proposed automated enhancement pipeline has revealed that there is good potential to detect defects of larger than 10 mm diameter and up to 5 mm depth across the entire test surface. This is the equivalent of possible metal loss detection as low as 37.5%. In fact, it is paramount to identify the occurring corrosion events as early as possible along the asset’s life-cycle in order to effectively mitigate the risk of accelerated corrosion and eventually the asset’s repair or disposal. The comparison of the unprocessed results of this work with the results of an example case in which heating long pulse was used as an excitation waveform revealed that the cooling stimulation can achieve an almost similar detection capability. The advantage of cold thermography for defect detection is that it can be used in a dynamic setup as presented. This allows the potential for in-situ applications. However, the inaccuracies caused by such a portable setup need to be compensated through sophisticated image processing techniques. In comparison, detection using heat sources generating long pulse e.g., halogen lamps can be more accurate due to the fixed positions of both the test sample and heat source. However, energy consumption is higher and the practicality is limited.

## 5. Future Work

There were, however, some challenges that potentially can pose inaccuracies in the results. One was the possible misinterpretation of a reference area as defective, particularly at a location between two or multiple neighbouring defects. This can be attributed to the complex lateral cold energy diffusion around the defects. As the next step towards the “characterisation” of defects, authors propose studying the pixel time-transient curves at various locations on each defect. The out-of-phase nature of pixels thermal evolutions as a result of dynamic experiment setup, as well as the undesirable blocking phenomena e.g., non-uniform cooling and lateral diffusion, will still remain challenges that need to be overcome.

## Figures and Tables

**Table 1:** AISI 1018 mild steel mechanical and thermal properties.

Chemical Composition		Mechanical and Thermal Properties	
Carbon, C	0.14–0.20%	Density	7.87×10^3^ (kg/m^3)^
Iron, Fe	98.81–99.26%	Tensile Strength, Yield	370 (MPa)
Manganese, Mn	0.60–0.90%	Thermal Conductivity	45–64.9 (W/m × K)
Phosphorous, P	≤0.040%	Specific Heat	510.7 (J/kg × K)

**Table 2:** The arrangement of depth and size of artificial defects.

Groups	A				B				C				D				E			
Sub-groups	1	2	3	4	1	2	3	4	1	2	3	4	1	2	3	4	1	2	3	4
D (mm)	20	15	10	5	20	15	10	5	20	15	10	5	20	15	10	5	20	15	10	5
d * (mm)	1	1	1	1	2	2	2	2	3	3	3	3	4	4	4	4	5	5	5	5

D: diameter; d: depth. * The depth is the distance measured from the test piece surface.

**Table 3:** The comparison of defect original and improved visibility and detection status.

Defect (d, D) mm	S1	S2	S3	S4	Note
A1 (1, 20)	√	√	√	√	S1: represents the status of defect visibility in the best original thermal image. S2: represents the status of defect visibility in the PCA of the original image (Figure 11c,d). S3: represents the status of the defect in the PCA of DRR images (Figure 11e,f). S4: represents the detection status of the defect in the GMM image (Figure 14e,f).
A2 (1, 15)	√	√	√	√	
A3 (1, 10)	√	√	√	√	
A4 (1, 5)					
B1 (2, 20)	√	√	√	√	
B2 (2, 15)	√	√	√	√	
B3 (2, 10)			√	√	
B4 (2, 5)					
C1 (3, 20)	√	√	√	√	
C2 (3, 15)	√	√	√	√	
C3 (3, 10)			√	√	
C4 (3, 5)					
D1 (4, 20)		√	√	√	
D2 (4, 15)		√	√	√	
D3 (4, 10)			√	√	
D4 (4, 5)					
E1 (5, 20)			√	√	
E2 (5, 15)			√	√	
E3 (5, 10)					
E4 (5, 5)					

## Data Availability

Not applicable.
